# Development of an Autonomous Unmanned Aerial Manipulator Based on a Real-Time Oriented-Object Detection Method

**DOI:** 10.3390/s19102396

**Published:** 2019-05-25

**Authors:** Shijie Lin, Jinwang Wang, Rui Peng, Wen Yang

**Affiliations:** 1 School of Electronic Information, Wuhan University, Wuhan 430072, China; linshijie@whu.edu.cn (S.L.); jwwangchn@whu.edu.cn (J.W.); pengrui@whu.edu.cn (R.P.); 2 State Key Laboratory for Information Engineering in Surveying, Mapping and Remote Sensing (LIESMARS), Wuhan 430079, China

**Keywords:** aerial manipulation, aerial system, deep learning

## Abstract

Autonomous Unmanned Aerial Manipulators (UAMs) have shown promising potential in mobile 3-dimensional grasping applications, but they still suffer from some difficulties impeding their board applications, such as target detection and indoor positioning. For the autonomous grasping mission, the UAMs need ability to recognize the objects and grasp them. Considering the efficiency and precision, we present a novel oriented-object detection method called Rotation-SqueezeDet. This method can run on embedded-platforms in near real-time. Besides, this method can give the oriented bounding box of an object in images to enable a rotation-aware grasping. Based on this method, a UAM platform was designed and built. We have given the formulation, positioning, control, and planning of the whole UAM system. All the mechanical designs are fully provided as open-source hardware for reuse by the community. Finally, the effectiveness of the proposed scheme was validated in multiple experimental trials, highlighting its applicability of autonomous aerial rotational grasping in Global Positioning System (GPS) denied environments. We believe this system can be deployed to many potential workplaces which need UAM to accomplish difficult manipulation tasks.

## 1. Introduction

During the past several decades, Unmanned Aerial Vehicles (UAVs) have shown surprising potential among numerous applications [1,2]. When mounted with different kinds of sensors, the UAVs can extend the sensing range and transform a static sensing task into a mobile sensing task. The UAVs’ advantages of freely using 3-dimensional (3D) space bring possibilities to many traditional tasks, like manipulation tasks in a warehouse or a factory.

Unmanned aerial manipulators (UAMs) are known as one specific type of UAVs equipped with one or multiple robotic arms and have attracted a lot of research interests in recent years [3]. One main advantage of a UAM is that it shows promising potential in transforming passive sensing missions into mobile 3D interactive missions, like grasping [4] and assembling [5]. Aerial maneuvering and hovering capabilities make it possible for a UAM to accomplish many kinds of missions that are difficult for human workers, such as grasping plastic bottle waste on a cliff. Furthermore, there are many places, like an Amazon warehouse, with the potential to deploy a UAM system for autonomous picking and placing. To fully utilize the space in a warehouse, goods can be placed at a relatively high place, which is difficult for human workers to reach. At this time, the UAM can give a lot of help. In future warehouses, there will be many UAMs flying simultaneously to sort the goods. Besides, places, like the Fukushima nuclear power plant in Japan, that are filled with obstacles and are hard for human workers to work inside are ideal places to deploy a UAM. Potential applications of UAMs can easily come from people’s imaginations and surely not be limited to the situations mentioned above, which is the reason why we believe UAMs have a bright future.

For autonomous aerial manipulation missions, building a controllable system is always the first step. However, many factors can influence the stability of the overall system, such as the change of Center of Gravity (CoG) generated by the movements of the robotic arm, the reaction force produced by the robotic arm, and the complex aerodynamics effects. Many efforts have been made to reduce these effects [6,7,8,9,10]. A comprehensive dynamic model of hexacopter with a robotic arm has been built in [6], analyzing the effects of CoG change and mass distributions. The kinematics and dynamics models of a quadrotor with n Degree of Freedom (DoF) robotic arm have been formulated in [7]. A Variable Parameter Integral Backstepping (VPIB) controller proposed in [8] can control a UAM with better performance than a Proportional Integral Derivative (PID) controller. With a movable compensation mechanism, a multilayer architecture controller that compensates for the internal and external effects, layer by layer, to control the UAM was presented [9]. To suppress the torque generated by the movements of the robotic arm, a novel mechanism with a simplified model has been adopted in [10]. For some scenarios where multiple UAMs are required to work at the same time, some swarm studies [11,12] are also worth considering.

Usually, human operators are unable to accurately control the UAM due to the point of view change and data transmission delay. Also, for the monocular camera, the lack of depth perception in teleoperation makes it hard for the operators to decide whether the object is appropriately put into the claw of robotic arm. Such inaccuracy causes the robotic arm to be difficult to align and grasp the objects. Therefore, it is better the UAM can work without relying on external control by human operators. However, if there is no external control by human operators, the UAM needs to recognize the objects by itself, meaning we need to give the UAM perception ability. Currently only a few works [13,14,15] considered robust visual perception aided autonomous grasping. An Image-Based Visual Servo (IBVS) was implemented in [13] to help to locate the position of the targeted object. Feature models were used in [14] to find the position of known targets. In [13], correlation filters were adopted to track targets.

In recent years, end-to-end object detection algorithms like [16,17,18] have shown surprising results. Faster R-CNN [16] is an end-to-end two-stage framework for object detection. It firstly uses the Region Proposal Network (RPN) to extract proposals which contain objects, then classifies and refines these proposals. You Only Look Once (YOLO) [17] is a one-stage object detector, and it is much faster than two-stage framework. Considering efficiency, SqueezeDet [18] fully utilized SqueezeNet [19], which is a small network but achieves AlexNet [20] level accuracy with 50x fewer parameters. And SqueezeDet achieves a good tradeoff between detection accuracy and efficiency.

**Figure 1:**
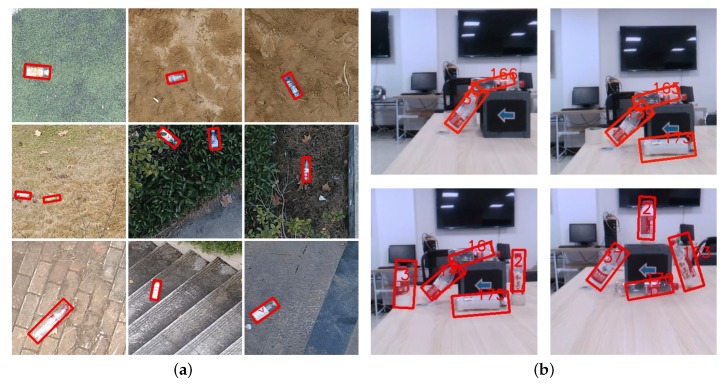
(**a**) Sample images in Unmanned Aerial Vehicle (UAV)-Bottle Detection (BD) dataset with plastic bottle groundtruth labeled by red box. (**b**) Detection results of Rotation-SqueezeDet with 2D box and rotation angle of multiple objects at the same time. Oriented angles of bottles are shown in the images.

However, attempts to directly apply these methods in the UAM system usually end with failure or unsatisfactory robustness. The reason lies in the fact that objects, like bottles, in the UAV perspective often appeared with arbitrary poses, as shown in Figure 1a, since the camera is tight-coupled and rotated with the UAV body. Moreover, most grasping missions need a target’s rotation angle for not touching the target when the end-effector is approaching. Furthermore, performance, like robustness and efficiency of the detection algorithms, plays an important role in grasping missions since it can bring more mobility to the UAM. However, current common oriented bounding boxes-based methods presented in [21,22] are designed for large-scale objects detection, like detecting a car in a large satellite image. Since the cars only make up a few pixels in a large satellite image, they need a much deeper convolution network to improve their accuracy. And this deep network cannot run in real-time performance even with an NVIDIA GTX1080 graphic card. Considering the size and weight requirement of a drone platform, the NVIDIA Jetson TX2 is the current optimal choice for mobile deep learning platforms. But methods like [21,22] are still unable to run onboard since both require more than 11G memory to load a deep convolution network, and the Jetson TX2 only has an 8G memory, let alone the real-time performance. Without a real-time performance, the grasping efficiency has been greatly reduced because the UAM needs to fly much slower to not pass the target.

In order to solve the problems mentioned above, we proposed Rotation-SqueezeDet, which can regress rotation angle and position in a 2-dimensional (2D) image in near real-time. Unlike the common horizontal bounding box descriptor $\left(c_{x},c_{y},w,h\right)$, where $\left(c_{x},c_{y}\right)$ is the center location, and *w* and *h* are the width and height of the bounding box, respectively, Rotation-SqueezeDet introduces a new θ term, and thus uses $\left(c_{x},c_{y},w,h,\theta \right)$ to describe object position and rotation angle in the 2D image. This not only makes the detection more robust since the bounding box included fewer background, but also provides the rotation angle of the target. By using an Intel RealSense D435 depth camera, the relative 3D distance of targets can be measured in the point cloud generated by registered depth image once the target is detected. Hence, a rotation-aware grasping for autonomous grasping is possible. A glimpse of detection results is shown in Figure 1b.

Now we want to deploy this detection in the UAM for autonomous grasping. However, the system mentioned in previous paragraphs have only considered neither the control problem or system compensate problem but an overall applicable indoor system. Usually, they test the system in an outdoor environment with strong Global Positioning System (GPS) signals for localization or use an expensive external visual caption system like VICON (www.vicon.com) to provide state estimation of the drone. Both of these two ways are hard to achieve in a real industrial environment. So, we adopted the lightweight Simultaneous Localization and Mapping (SLAM) system VINS-Mono [23] for indoor state estimation, giving our system the ability to fly indoors with only an inexpensive camera. Furthermore, based on the Rotation-SqueezeDet, we designed and implemented a complete indoor, workable UAM system.

The main contributions of this paper can be summarized in two folds. First, the Rotation-SqueezeDet method was proposed. This method can run on Jetson TX2 in near real-time and enable successful rotation-aware grasping. Moreover, we believe that this method will be suitable in not only the aerial grasping but also in more general missions. Second, we have designed and assembled the whole UAM platform and fully tested this system in an indoor environment. The system is affordable since it costs less than $2300 USD and can fly without relying on expensive visual motion caption system. We believe this system can be adopted to many potential workplaces, like Amazon warehouses, which need UAMs to accomplish difficult manipulation tasks. All mechanical structures are provided as open-hardware for reuse by the community (https://github.com/eleboss/UAMmech).

The rest of this paper is organized as follows. Section 2 describes the design and details of the overall system. Section 3 describes the formulation, control, and planning of the UAM system. Section 4 describes the complete vision system including Rotation-SqueezeDet. Section 5 experimentally demonstrates the system including autonomous grasping and vision system performance in an open dataset. Finally, the conclusion and future work are presented in Section 6.

## 2. System Description

### 2.1. Notation

**Figure 2:**
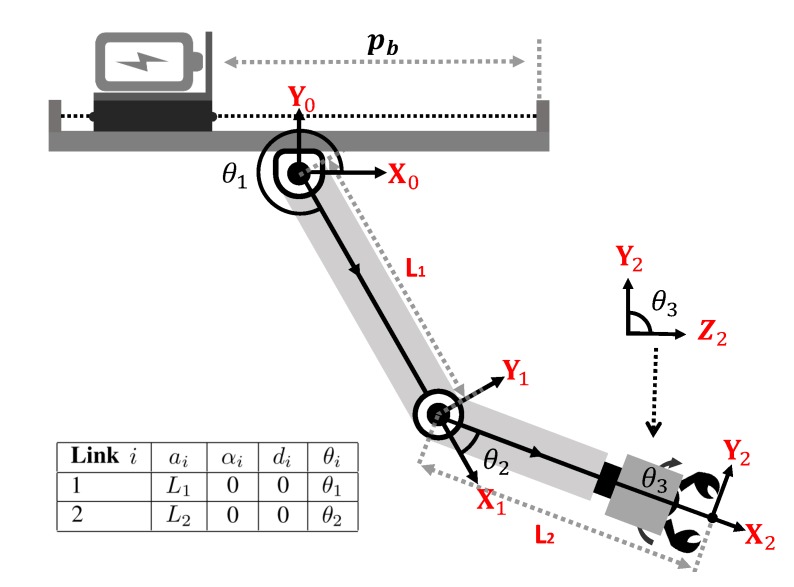
The coordinate system and Denavit–Hartenberg (D–H) parameters of the robotic arm.

The East, North, and Up (ENU) coordinate system are used as a world-fixed inertial frame corresponding to $\left\{x_{w},y_{w},z_{w}\right\}$. Following the definition of well known Denavit–Hartenberg (D–H) parameters [24], the link frame of Link *i* is defined as the $\left\{x_{i},y_{i},z_{i}\right\}$. Especially, we define $\left\{x_{0},y_{0},z_{0}\right\}$ as the fixed arm frame. The definition of the link frame is detailed in Figure 2 and the table in the bottom left gives the D-H parameters of the robotic arm. The body frame is assumed to be the geometrical center of the UAM denoted as $\left\{x_{b},y_{b},z_{b}\right\}$. $G_{x}$, $G_{y}$, $G_{z}$ indicate the CoG of the UAM in body frame $\left\{x_{b},y_{b},z_{b}\right\}$. (ϕ,θ,ψ) indicate the roll-pitch-yaw Euler angels. $\left\{x_{t},y_{t},z_{t}\right\}$ define the detected targets in the RealSense D435 camera frame.

### 2.2. Hardware

**Figure 3:**
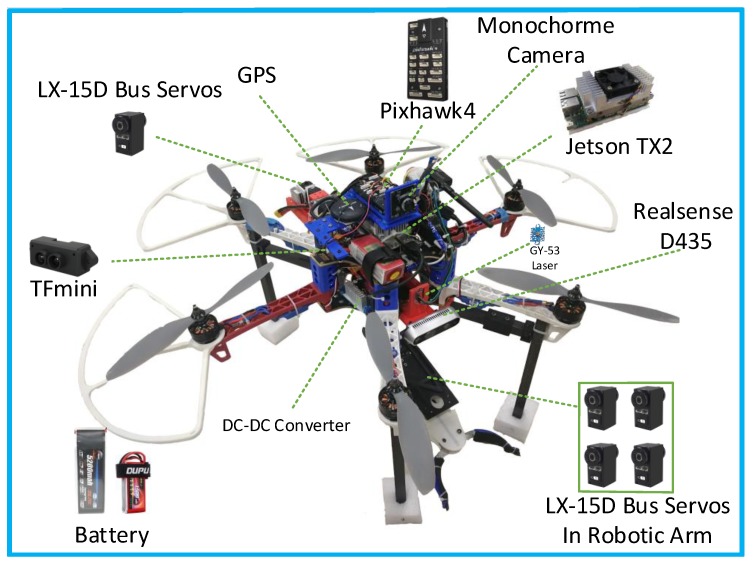
Hardware components of the Unmanned Aerial Manipulator (UAM).

In this work, a modified DJI hexacopter frame F550 (www.dji.com/flame-wheel-arf/feature) was adopted. The hardware components are shown in Figure 3.

The propulsion module of the UAM was composed of Sunnysky (www.rcsunnysky.com) x3108s motor, Hobbywing (www.hobbywing.com) platinum Electronic Speed Controller (ESC), and a 10-inch propeller. The Pixhawk4 (www.holybro.com/product/55) autopilot with PX4 (https://github.com/PX4/Firmware) V1.8.0 flight stack and NVIDIA Jetson TX2 (developer.nvidia.com/embedded/buy/jetson-tx2) were placed at the top of the UAM as the main computing devices. A global shutter monochrome camera (www.jinyandianzi.com) was tight-coupled with the Pixhawk using a 3D-printed anti-vibration damping plate. A Benewake TFmini (www.benewake.com/tfmini.html) laser rangefinder was chosen for being cheap, lightweight, and with up to 12 m maximum detection range, and was mounted downward facing to provide altitude feedback. An Intel RealSense D435 (www.realsense.intel.com/stereo) camera was mounted at the middle of the drone, facing forward to find the targets.

We built a Displacement Compensation System (DCS) that can move counterweight to align the CoG and thus improves the stability of the total system. The DCS was mounted in the middle of UAM and made by 3D printed Poly Lactic Acid (PLA) material, including tow rails, a slide table, and a bus servo to provide drive force. The LEBOT (www.lobot-robot.com) LX-15D serial bus servos were chosen for being budget-friendly, lightweight, and having multiple extra structures to facilitate the installation. Another significant advantage of the bus servo is it can largely reduce the wiring complexity and control difficulty. So we could only use one serial port in the Jetson TX2 to control all servos. While the LX-15D servo can only provide $240^{\circ }$ feedback, we used a short-range Time of Flight (ToF) laser rangefinder GY-53 to provide the battery position feedback.

A 5200 mAh 4S-35C battery weighted 0.525 kg was used for providing enough power to the propulsion system and as a counterweight for the DCS. And this battery could sustain the flight time around 10 min. Another 1500 mAh 3S-30C weight 0.138 kg battery was used for providing power to the robotic arm and computing facilitates.

Drive forces of the robotic arm were also provided by LX-15D servos. The robotic arm had 3-DoF and could grasp objects by the end-effector. The first 2-DoF provided the robotic arm mobility to move at the planar 2D plane. The last DoF enabled a rotational grasping by cooperating with the vision system. The last DoF was of vital importance for the oriented object grasping, since the unrotated grasping could easily tip the object.

The total takeoff weight of the UAM was about 4.08 kg. Thanks to the carbon fiber material and PLA material, the robotic arm was only weighted 0.459 kg and had 43 cm extended range.

### 2.3. Software Architecture

**Figure 4:**
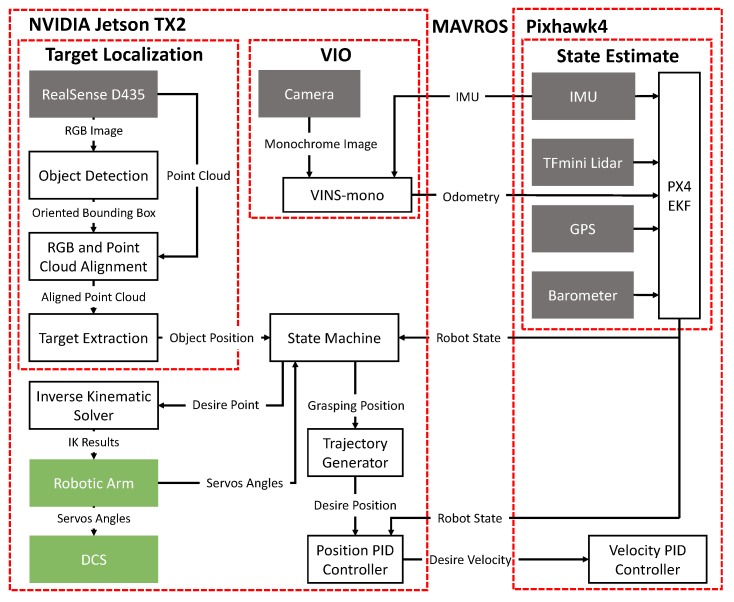
An overview of the software architecture. PID = Proportional Integral Derivative; IMU = Inertial Measurement Unit.

The software runs on two main processors: Pixhawk4 and Jetson TX2, and all processes are running onboard. Figure 4 gives an overview of the system software architecture. Note that the Jetson TX2 is overclocked to run at the maximum clock rate and unlocked two external cores to generate more computing power.

The Robot Operating System (ROS) [25] is a pseudo-operating system which allows developers to work cooperatively by following its running mechanism. Modules related to state estimation and flying control are running on the Pixhawk4 and exchange data with the Jetson TX2 by MAVROS (https://github.com/mavlink/mavros). Visual Inertial Odometry (VIO) and object detection algorithms are running on the Jetson TX2. A state machine is adapted to set the robotic arm motion and UAM waypoint. So, the grasping position $\left(x_{w}^{s},y_{w}^{s},z_{w}^{s}\right)$ of the UAM can be given by:

(1) $$\begin{array}{c} \left[\begin{array}{c} x_{w}^{s}\\ y_{w}^{s}\\ z_{w}^{s}\\ 1 \end{array}\right]=\left[\begin{array}{c} x_{w}\\ y_{w}\\ z_{w}\\ 1 \end{array}\right]+T_{B}^{W}T_{C}^{B}\left[\begin{array}{c} x_{t}\\ y_{t}\\ z_{t}\\ 1 \end{array}\right]-T_{B}^{W}T_{0}^{B}\left[\begin{array}{c} x_{0}^{g}\\ y_{0}^{g}\\ 0\\ 1 \end{array}\right] \end{array},$$

where $T_{B}^{W},T_{0}^{B},T_{C}^{B}\in R^{4\times 4}$ are the homogeneous transformation matrix, $T_{0}^{B}$ transforms the fixed arm frame to body frame, $T_{C}^{B}$ transforms the camera frame to the body frame, $T_{B}^{W}$ transforms the body frame to the world-fixed frame, and $\left(x_{0}^{g},y_{0}^{g}\right)$ is the grasping point.

The standard cascaded position-velocity control of the hexacopter is well implemented inside the Pixhawk4 fly controller. More detailed information can be found at this link (https://dev.px4.io/en/flight_stack/controller_diagrams). We adopted this control strategy and well tuned its inside parameters to get a robust performance.

### 2.4. State Estimation

State estimation is the foundation of our system, as it provides crucial information to help other parts to achieve the best performance. In this work, we use the Pixhawk4 built-in Extended Kalman Filter (EKF) to fuse multiple sensors’ feedback for state estimation.

GPS usually fails to provide global positioning feedback when in an indoor environment. Hence, in order to fly indoors without using expensive visual motion caption system, we integrated the VINS-mono VIO to provide the local position feedback, and it can provide the highest level of accuracy and robustness compared with multiple VIO [26]. The VINS-mono runs at 10hz with loop-closure and the output is rotated to ENU world-fixed frame denoted $\left(x_{w}^{v},y_{w}^{v},z_{w}^{v}\right)$. For the VINS-mono, we used the Inertial Measurement Unit (IMU) in the Pixhawk4 as the inertial input, and a monochrome global shutter camera ran at 640×400 resolution and 90 Frames Per Second (FPS) to provide clear images as visual input. To reduce drifting, the monochrome camera and Pixhawk4 are tight-coupled by the 3D printed structures, the camera intrinsic matrix and camera to IMU transformation parameters are carefully calibrated by using kalibr [27]. Due to the computation limitation and data transmission delay, the output of VINS-mono runs in Jetson TX2 has about 140ms delay compared with current IMU output. In order to synchronize these outputs, we first calculate the velocity of VINS-mono estimation $\left({\dot{x}}_{w}^{v},{\dot{y}}_{w}^{v},{\dot{z}}_{w}^{v}\right)$, then apply some random movements to the UAM, so the delay time can be found by comparing $\left({\dot{x}}_{w}^{v},{\dot{y}}_{w}^{v},{\dot{z}}_{w}^{v}\right)$ with the Pixhawk4 velocity estimation. Finally, $\left(x_{w}^{v},y_{w}^{v},z_{w}^{v}\right)$ is used as external vision aid of the Pixhawk4 onboard EKF to give 100hz state estimation.

For robust flying, the main altitude feedback is not given by the VINS-mono but the TFmini rangefinder. The total delay of TFmini measurement is about 30 ms.

## 3. Robotic Arm & DCS

### 3.1. Robotic Arm Motion Planning

In order to find the workspace of robotic arm, the forward kinematics of a 3DoF robotic arm is given by the following equations:

(2) $$\begin{array}{c} x_{0}=L_{1}\cos\theta_{1}+L_{2}\cos\left(\theta_{1}+\theta_{2}\right)\\ y_{0}=L_{1}\sin\theta_{1}+L_{2}\sin\left(\theta_{1}+\theta_{2}\right)\\ \theta_{3}=\theta , \end{array}$$

where $L_{1}$, $L_{2}$ are the lengths of the first and the second links. $\theta_{1}$, $\theta_{2}$, $\theta_{3}$ are the rotation angles of each joint, and $\left(x_{0},y_{0}\right)$ is the point in arm fixed frame. Since the angle $\theta_{3}$ is only related to the target rotation angle θ, the workspace of our robotic arm is equal to a planar 2DoF robotic arm model. The table presented in Figure 2 gives a clear D-H parameters definition of the robotic arm.

**Figure 5:**
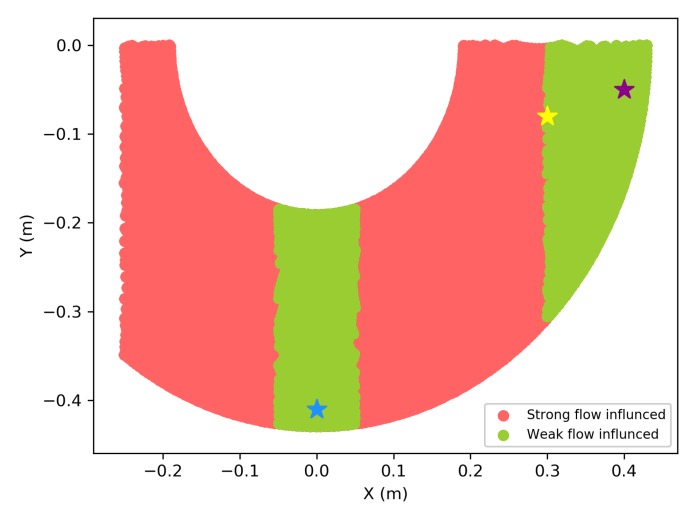
Workspace of robotic arm printed by using forward kinematics considered flow influence.

When applying the UAM into real-world scenes, the flow generated by rotors can blow the lightweight targets away, like empty plastic bottles, and it is hard to predict whether an object can be easily blown away by the downward flow or not. Hence, a safety grasping action is better to be taken under weak flow influenced conditions. High-fidelity Computational Fluid Dynamics (CFD) simulation results of many different UAVs have been presented in [28]. From the observation of these results, the flow generated by each UAV’s rotor is decreasing rapidly in the outer-wing area. Inspired by this observation, we planned the robotic arm to grasp in a weak flow area. Based on the flow measurements described in Section 5.2 and the actual mechanical movement range of robotic arm, the actual workspace is given in Figure 5, the green points indicate weak flow influenced area, and red points indicate strong flow influenced area. The blue star point is the dropping point and the purple star point is the grasping point $\left(x_{0}^{g},y_{0}^{g}\right)$. The robotic arm holds at the yellow star point $\left(x_{0}^{f},y_{0}^{f}\right)$ during flight. And all these points can be redefined depending on the applications.

We used Inverse Kinematics (IK) to solve the desired rotation angle of each joint. Assuming the robotic arm is planning to move to $\left(x_{0},y_{0}\right)$ point, the IK result is given by:

(3) $$\begin{array}{c} \theta_{2}=\pm \arccos\left(\frac{{x_{0}}^{2}+{y_{0}}^{2}-{L_{1}}^{2}-{L_{2}}^{2}}{2L_{1}L_{2}}\right), \end{array}$$

Here, $\theta_{2}\in \left[0,\pi \right]$ is the downward elbow solution, and $\theta_{1}$ can be derived by:

(4) $$\begin{array}{c} \theta_{1}=\arctan\left(\frac{{x_{0}}^{2}}{{y_{0}}^{2}}\right)-\arccos\left(\frac{{x_{0}}^{2}+{y_{0}}^{2}+{L_{1}}^{2}-{L_{2}}^{2}}{2L_{1}\sqrt{{x_{0}}^{2}+{y_{0}}^{2}}}\right). \end{array}$$

### 3.2. CoG Compensation

When the UAM is static on the ground and the robotic arm holds static at $\left(x_{0}^{f},y_{0}^{f}\right)$, a symmetry placement design is utilized to make sure the $G_{x}$ and $G_{y}$ are fitted with the geometry center.

However, movements of the robotic arm can change the $G_{x}$. For the dynamic $G_{x}$ alignment, we adapted the strategy presented in [9] called DCS, moving the battery as a counterweight since its weight can provide sufficient compensation in the relatively short moving distance.

CoG transformation of Link *i* and the end-effector payload from link frame to the body frame are given by:

(5) $$\begin{array}{c} \left[\begin{array}{c} x_{bi}^{g}\\ y_{bi}^{g}\\ z_{bi}^{g}\\ 1 \end{array}\right]=T_{0}^{B}T_{i}^{0}\left[\begin{array}{c} x_{i}^{c}\\ y_{i}^{c}\\ z_{i}^{c}\\ 1 \end{array}\right], \end{array}$$

where $T_{i}^{0},T_{0}^{B}\in R^{4\times 4}$ are the homogeneous transformation matrices, $T_{0}^{B}$ transforms from fixed arm frame to body frame, and $T_{i}^{0}$ transforms from each link frame to the fixed arm frame. ($x_{i}^{c}$, $y_{i}^{c}$, $z_{i}^{c}$) is the CoG position of Link *i* in the fixed arm frame. ($x_{bi}^{g}$, $y_{bi}^{g}$, $z_{bi}^{g}$) is the CoG position of Link *i* in body frame, and here i=3 indicates the grasped object.

To align the $G_{x}$ at geometry center, a linear slider is designed to move the battery and the position of the battery $p_{b}$ in the body frame can be calculated by:

(6) $$\begin{array}{c} p_{b}=\frac{\sum_{i=1}^{3}m_{i}x_{bi}^{g}}{m_{b}}, \end{array}$$

where $m_{i}$ is the mass of Link *i*, $m_{b}$ is the mass of battery.

The displacement compensation plays a key role in stabilizing the UAM. Without the DCS, the change of CoG can easily make aside rotor reach the maximum thrust, leading to it being unstable. And this method worked well in our system. To guarantee the compensation performance, we chose a reasonable range of rotation speed of each joint in a robotic arm to make sure it did not exceed the maximum compensation speed of the linear slider.

### 3.3. Control Strategy of Robotic Arm

**Figure 6:**
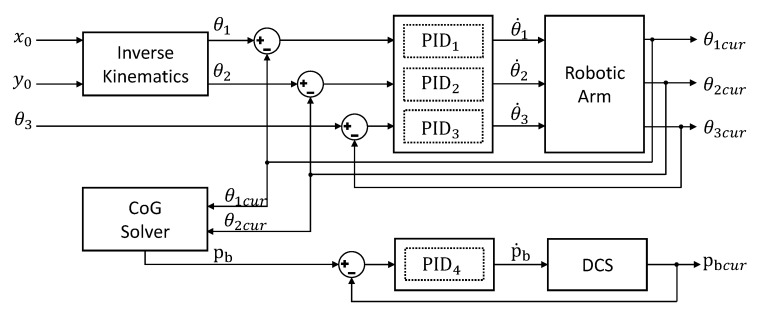
Control diagram of the Displacement Compensation System (DCS) and robotic arm. CoG = Center of Gravity.

The total control diagram of the robotic arm and DCS are shown in Figure 6. Here, ${\dot{\theta }}_{1},{\dot{\theta }}_{2},{\dot{\theta }}_{3},{\dot{p}}_{b}$ is the rotation speed of the servos and the $\theta_{1cur},\theta_{2cur},\theta_{3cur},p_{bcur}$ indicate the current feedback. Three PID controllers are adapted to control the robotic arm. Another PID controller uses the position of the battery $p_{b}$, detected by a laser rangefinder, to control the linear slider. All PID parameters are well tuned to guarantee stable and smooth control.

## 4. Vision System

In this section, we introduce the vision system, including the light and fast oriented-object detection model called Rotation-SqueezeDet and the target localization framework based on Rotation-SqueezeDet detection results, and point clouds from the depth camera.

Our proposed model is inspired by SqueezeDet [18] and Rotation Region Proposal Networks (RRPN) [21]. As the former, SqueezeDet is a single-pass detection pipeline combining bounding box localization and classification by a single network. It appears to be the smallest object detector by virtue of a powerful but small backend network of SqueezeNet [19,29]. As the latter, RRPN is based on Faster R-CNN [16], but RRPN can detect oriented objects. The difference between Faster R-CNN and RRPN is described as below. In Faster R-CNN, the Region of Interests (RoIs) are generated by RPN, and the RoIs are rectangles which can be written as $R=\left(x_{min},y_{min},x_{max},y_{max}\right)=\left(c_{x},c_{y},w,h\right)$. These RoIs have regressed from *k* anchors which are generated by some predefined scales and aspect ratios. Then these RoIs will be feed into RoI Pooling layer and some fully connected layers to obtain horizontal bounding boxes. However, in RRPN, instead, it uses Rotation anchors (R-anchors) and rotation RoI Pooling, which brings the ability to predict oriented bounding boxes denoted as $R=(c_{x},c_{y},w,h,\theta )$.

Object detection algorithms like Faster R-CNN have high accuracy but slow processing speed and large storage requirement. Moreover, RRPN is slower than Faster R-CNN, it takes twice as much time as the Faster-RCNN [21]. Thus, RRPN does not meet our run-time requirement. Considering a comparable accuracy and run-time on Jetson TX2, SqueezeDet is a suitable choice, it can run about 45FPS with 424×240 pixels image on Jetson TX2, and is easy to train. However, SqueezeDet cannot predict the θ of the rotated object because it uses horizontal bounding boxes. So, we designed a model which can generate oriented bounding boxes based on SqueezeDet and named Rotation-SqueezeDet, and it can run on Jetson TX2 in near real time.

### 4.1. Network Architecture

**Figure 7:**
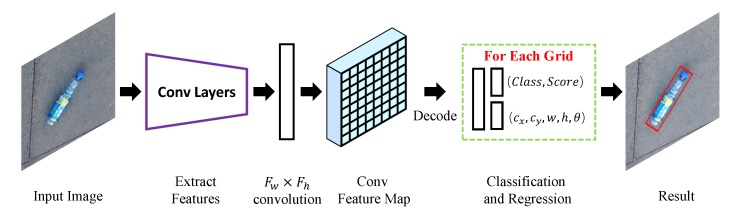
Rotation-SqueezeDet’s detection pipeline.

The overall model of Rotation-SqueezeDet is illustrated in Figure 7. In this model, a convolutional neural network, SqueezeNet V1.1, first takes an image as input and extracts a low-resolution, high dimensional feature map from the image. Then the feature map is fed into the $F_{w}\times F_{h}$ convolutional layers to compute oriented bounding boxes at each position of conv feature map. Next, each oriented bounding box is associated with (5+C+1) values, where 5 is the number of bounding box parameters, *C* is the number of classes, and 1 is the confidence score. And each position on the convolution feature map computes K×(5+1+C) values that encode the bounding box predictions. Here, *K* is the number of R-anchors, each R-anchor can be described by 5 parameters as $\left(c_{x}^{a},c_{y}^{a},w^{a},h^{a},\theta^{a}\right)$, $\left(c_{x}^{a},c_{y}^{a}\right)$ are R-anchor’s center on image, $w^{a},h^{a},\theta^{a}$ are the width, height and angle of R-anchor, respectively.

(7) $$\begin{array}{c} v_{x}=\frac{c_{x}-c_{x}^{a}}{w^{a}},v_{y}=\frac{c_{y}-c_{y}^{a}}{h^{a}},\\ v_{w}=\log\frac{w}{w^{a}},v_{h}=\log\frac{h}{h^{a}},v_{\theta }=\theta -\theta^{a}+k\pi , \end{array}$$

where $\left(c_{x},c_{y},w,h,\theta \right)$ are parameters describe the predicted oriented bounding box, $\left(c_{x}^{a},c_{y}^{a},w^{a},h^{a},\theta^{a}\right)$ are parameters describe the R-anchor, and here k∈Z to ensure θ∈[0,π).

### 4.2. Oriented IoU Computation

To efficiently predict the rotation angle of objects, we introduce a parallel Intersection over Union (IoU) computing method. First, we attempt to calculate the IoU using the OpenCV’s functions *rotatedRectangleIntersection* and *contourArea* directly. However, the efficiency of these functions remains poor because they cannot compute parallel. Thus, we use a simple and efficient method to approximately compute the IoU in parallel, which is to use the angle deviation of two oriented bounding boxes. The approximate IoU [30] can be computed by:

(8) $$\begin{array}{c} {\mathrm{IoU}}^{★}=\mathrm{IoU}*\mathrm{abs}\left(1-\frac{\theta_{1}^{b}-\theta_{2}^{b}}{\pi }\right), \end{array}$$

where $\theta_{1}^{b}$ and $\theta_{2}^{b}$ are the rotation angle of two oriented bounding boxes, and IoU is computed by treating oriented bounding boxes as horizontal bounding boxes.

### 4.3. R-Anchors’ Selection

The R-Anchors are different from the horizontal anchors. And for the R-Anchors’ selection, we use a K-means based method described in [31] to select R-anchors’ *w* and *h* to match the data distribution, we set *k* as 9 in K-means and treat objects’ angle distribution as a uniform distribution, i.e., we set R-anchors’ angle as $\left\{0,\frac{\pi }{9},\frac{2\pi }{9},\frac{3\pi }{9},\frac{4\pi }{9},\frac{5\pi }{9},\frac{6\pi }{9},\frac{7\pi }{9},\frac{8\pi }{9}\right\}$. Therefore, there are 81 anchors at each conv feature map position.

### 4.4. Object Localization

To acquire the real world position of the target, we use the RGB-D camera to detect and locate the target. First, the aligned color image and point clouds can be obtained by aligning RGB image and depth image in the same coordinate system. Next, the subarea of the total point clouds containing location information of the target which can be extracted from the whole point clouds by utilizing the detection result $\left(c_{x},c_{y},w,h,\theta \right)$. After that, we use a small central subarea of target’s point clouds to calculate its real-world position $\left(x_{t},y_{t},z_{t}\right)$ in the camera frame given by:

(9) $$\begin{array}{c} \left(x_{t},y_{t},z_{t}\right)=\left(\frac{1}{L}\sum_{i=0}^{k^{2}}X_{p}^{i},\frac{1}{M}\sum_{i=0}^{k^{2}}Y_{p}^{i},\frac{1}{N}\sum_{i=0}^{k^{2}}Z_{p}^{i}\right), \end{array}$$

where $\left(X_{p},Y_{p},Z_{p}\right)$ are the coordinates of the point clouds set of the center subarea of target’s bounding box, its superscript indicates the $i^{th}$ point value started from top left corner. L,M,N are the valid points number of $X_{p},Y_{p},Z_{p}$, respectively. The central subarea of target’s point clouds can be written as $\left(c_{x},c_{y},k,k\right)$, the k×k is the size of the selected central subarea of target’s point clouds, which can be calculated by:

(10) $$\begin{array}{c} k=\left\{\begin{array}{cc} 5 & \mathrm{if}\min(w,h)>5\\ \min(w,h) & \mathrm{otherwise} \end{array}.\right. \end{array}$$

Here, we set *k* as 5 to reduce the computing burden. Finally, the parameters of position and rotation angle of a target can be written as: $\left(x_{t},y_{t},z_{t},\theta \right)$, θ is the rotation angle of target’s anchor.

## 5. Experiments

The experimental setup is mentioned in Section 2. During the flight tests, all data are logged onboard with no external data transmission.

### 5.1. Vision System Results

We trained and evaluated our vision system based on our pervious work UAV-Bottle Detection (BD) [32], a bottle image dataset under UAV perspective. It contains about 34,791 object instances in 25,407 images labeled by oriented bounding boxes. For training and evaluating our model, 64% of the images were randomly selected as the training data, 16% as validation data, and the rest 20% as the testing data.

All object detection experiments were implemented on TensorFlow [33]. We used the pertained model, SqueezeNet v1.1, to initialize the network. And the system was trained 100k steps with a batch size of 20 and a learning rate of 0.01. Besides, weight decay and momentum were 0.0001 and 0.9, respectively. The optimizer was *MomentumOptimizer*.

**Figure 8:**
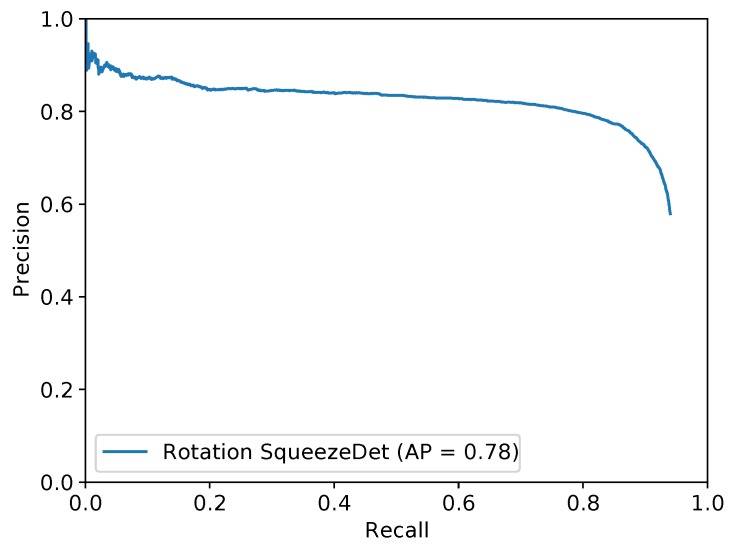
Average Precision (AP) results of Rotation-SqueezeNet validated in UAV-BD.

**Figure 9:**
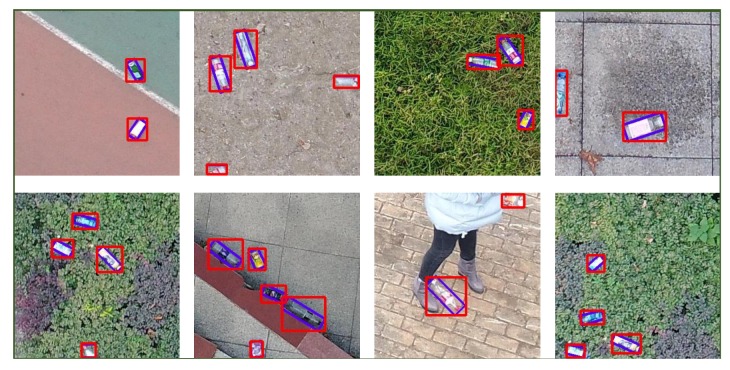
Detection results of the proposed detection algorithm in UAV-BD dataset. We demonstrate results in subfigures with varied backgrounds like grassland, playground and ground surface. Noticed that our algorithm could give the common bounding box in red and oriented bounding box in blue at the same time.

As shown in Figure 8, the Average Precision (AP) of Rotation-SqueezeDet on UAV-BD is about 78.0% when IoU=0.5. In Figure 9, we visualize the detection results in UAV-BD dataset. Our algorithms can be given two results; first is the common horizontal bounding box, marked by red color, without any rotation, and second is the oriented blue bounding box inside the red bounding box. We can clearly see that the blue boxes are more accurate than the red boxes since it contains fewer background pixels and locates the object more precisely.

To analyze the performance of time consumption, we deployed Rotation-SqueezeDet on three different platforms and recorded the average processing time with 424×240 pixels color image. The detailed information of the deployed platform is shown in Table 1, and the results are shown in Table 2. From Table 2, we can see that our algorithm has achieved near real-time performance in the Jetson TX2 and real-time performance on both the Laptop and Server platform. Notice that our algorithm could run up to 111 FPS with a Titan Xp GPU, and it can save a lot of time during the training of Rotation-SqueezeDet.

The time consumption of Rotation-SqueezeDet is mainly coming from two parts. The first part is the processing of detection model based on SqueezeNet. The second part is the processing of Non-maximum Suppression (NMS). The processing speed of detection model is related to GPU, and the processing speed of NMS is related to CPU. So, using a more powerful GPU can speed up the detection model processing, and a more powerful CPU will process the MNS much faster.

### 5.2. Flow Distribution Validation

**Figure 10:**
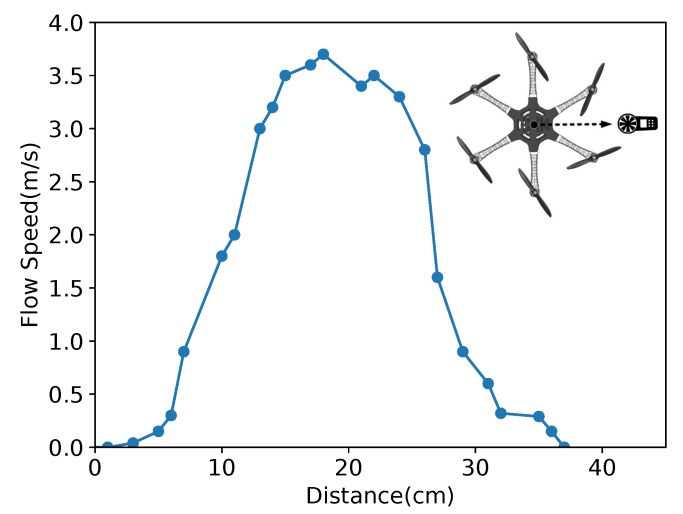
The flow speed measured by digital anemometer and the subfigure in the top right corner is the path of the anemometer during measurement.

In order to give a rough parameter estimation for the planning mentioned in Section 3, we disarmed the UAM and keep it flying at a certain height about 1.5 m above the ground. Then we used a Smart AS856 (en.smartsensor.cn/products_detail/productId=248.html) anemometer to measure the downward flow distribution. Since the flow is highly complex, we cannot give a very accurate flow distribution by just using an anemometer; thus, we only measured a 1D flow distribution to give a rough estimation for the robotic arm planning. The moving path of the anemometer is given in the top right corner of Figure 10 and the vertical distance from the anemometer to the rotor is about 16cm to represent the overall rough distribution below the UAM.

Following this path, we recorded the average value of 5 measurements at one point and drew the curve in Figure 10. The horizontal axis is the distance from the central position of the UAM body to the outside following this path. From the observation of Figure 10, the flow speed is decreasing rapidly at about 8 cm and 30 cm, and reaching the top speed at about 21 cm which is the underside of rotors. The 8 cm is close to the inside of the UAM, and the 30 cm is close to the outside of the UAM. So, the flow speed is relatively weak when at the central position and outside position of the UAM.

### 5.3. Aerial Robotic Arm Moving Test

**Figure 11:**
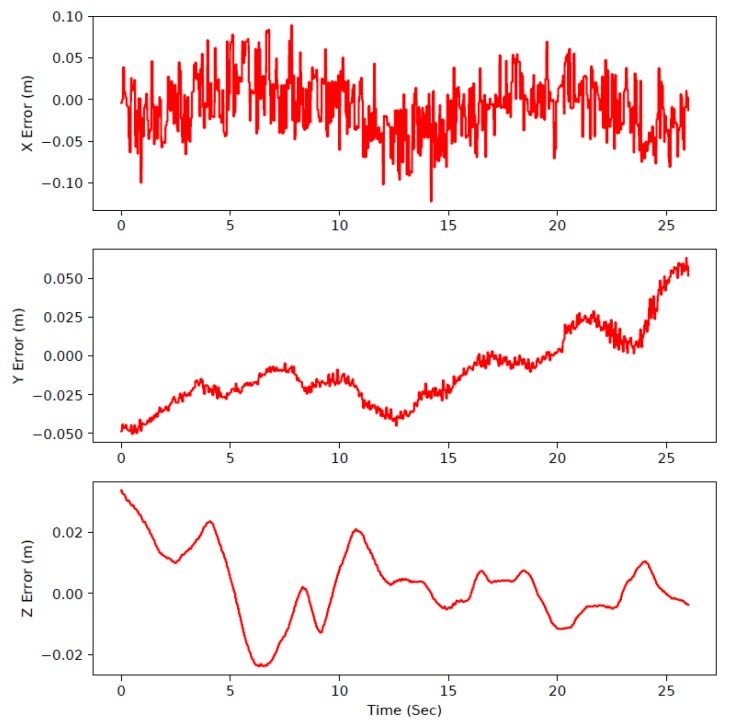
Control errors history during the whole aerial robotic arm moving.

To evaluate the stability and controllability of our system, the UAV was programmed to hovering at a certain point. In Figure 11, the control errors of the system during this flight were logged and presented. After finishing the takeoff procedure, the robotic arm will automatically move to multiple points and rotate the end-effector $90^{\circ }$.

**Figure 12:**
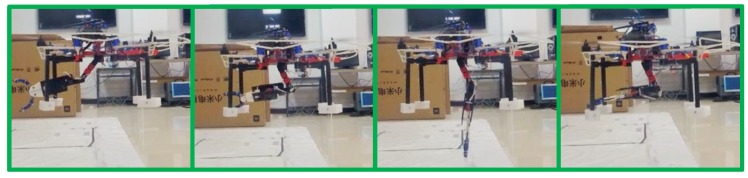
Snapshots of the robotic arm aerial moving experiments. Many movements are carried out during this flight to test the system. Each subfigure indicates a kind of movement of the robotic arm using in this experiment.

The DCS was activated in this flight, moving the battery to compensate for the change of CoG generated by movements of the robotic arm. The standard deviation of control error in the world-fixed frame is about 3.64 cm in x axis, 2.37 cm in y axis and 1.16 cm in in z axis, which indicated our system can keep hovering at the certain point whether the robotic arm is moving or not. We noticed that during the whole test, the error increased at several points including 5, 10 and 22 s, respectively. This was caused by the moving of the robotic arm, but our system could always stabilize itself. Figure 12 shows some snapshots of the robotic arm aerial moving experiments.

### 5.4. Autonomous Grasping

**Figure 13:**
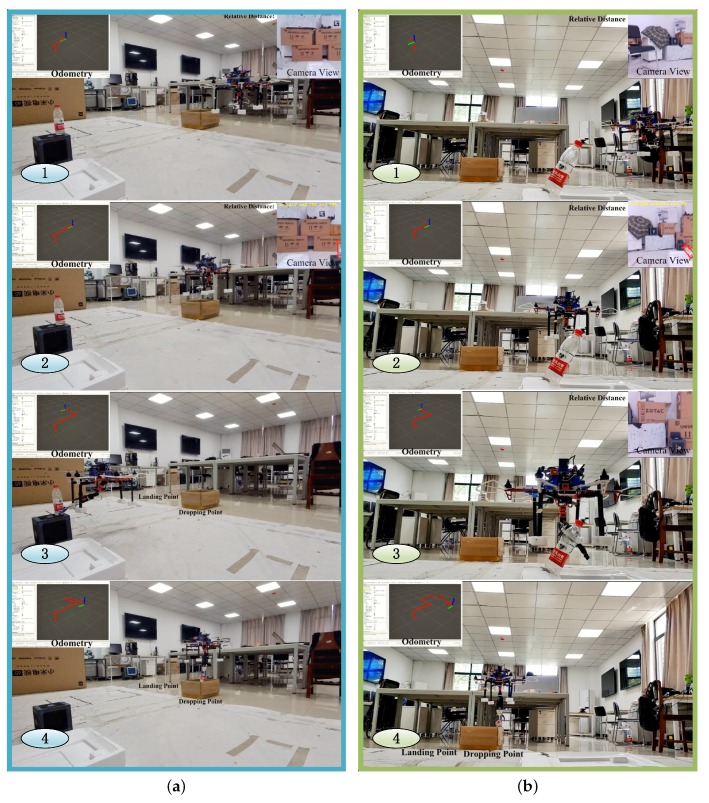
Snapshots of autonomous grasping experiments. The number in subfigures indicate selected moments: ① indicated the UAM is searching specific target; ② indicated the UAM detected the target and gives its relative position; ③ indicated the UAM hovering at the grasping position, and the end effector is about to grasp the bottle. ④ indicated the UAM dropping the grasped target at the dropping points. (**a**) Autonomous grasping of a bottle placed vertically; (**b**) autonomous grasping of a bottle placed obliquely.

Autonomous grasping experiments with objects placed vertically and obliquely have also been conducted. Figure 13 presents the key steps of these two grasping experiments. The subfigures were taken at the specific moments ordered by the number in the bottom left corner: ① indicated the UAM is searching specific target. Here, we used the plastic bottle as the target; ② indicated the UAM has detected the target and given its relative position. The UAM will then align its position to the grasping position; ③ indicated the UAM hovering at the grasping position and the end effector is about to grasp the bottle. ④ indicated the UAM dropping the bottle at the dropping point. The odometry given by the onboard EKF in 100 hz rates were simultaneously presented in the top left corner, showing our system can run indoors without using any external visual motion caption system. The detection results and the calculated relative distance of the target objects were also simultaneously presented in the top right corner during searching.

In Figure 13a, the target was vertically placed at the top of a cube. By comparing images captured at ① and ②, it is easily to know the bottle is not shown in ① but detected in ②. The UAM was using a constant speeded to search in ①, and speeded up to get close to target after ②. The end-effector successfully approached and grabbed the target in ③ verified our methods worked well, and the plastic bottle was not flipped by the downward flow. In ④, the UAM can automatically drop the target at the dropping point, which indicates our system is capable of automatically finishing the whole grasping task.

In Figure 13b, the procedures were basically the same as mentioned above. What is different is that we placed the empty bottle with about $45^{\circ }$ rotation. ② gave the detected target with its relative position and rotated angle. In ③, the end-effector used the rotation information of the target to successfully grasp the target.

These experimental results have shown that our system can accomplish an autonomous grasping mission.

## 6. Conclusions

In this paper, we developed an approach to enable a UAM to grasp oriented objects. The key challenges included detecting and locating objects in UAM perspective, CoG compensation, the flow influence to the objects, and indoor positioning of UAM system, all of which made the autonomous grasping mission very difficult. We showed the effectiveness of our approach in real tests with the ability to detect, locate, and grasp objects with arbitrary poses in GPS-denied environments without relying on the visual motion caption system. We believe that the proposed solution, both in terms of hardware and algorithms, will be useful in not only the aerial grasping missions but also in general grasping missions since the vision system can be applied to any kind of robotic arm. Future work will be set out to investigate how to estimate the 6D pose of objects in real-time. We will also improve the stability by using the servos with torque feedback and exploring a more advanced controller suit for the UAM.

## Figures and Tables

**Table 1:** Details of platforms using in the processing time test.

	Laptop	Server	Jetson TX2
Central Processing Unit (CPU)	i7-6700HQ @ 2.6 GHz	E5-2620 V4 @ 2.10 GHz	Cortex-A57 @ 2 GHz
Graphics Processing Unit (GPU)	GTX 960M	GTX Titan Xp	256-core Pascal

**Table 2:** Processing time of Rotation-SqueezeDet in different platform.

	Laptop	Server	Jetson TX2
Model Processing Time (s)	0.017	0.005	0.0035
NMS Processing Time (s)	0.003	0.004	0.006
Overall Processing Time (s)	0.020	0.009	0.041
Overall FPS	50	111	24

## Abbreviations

The following abbreviations are used in this manuscript:

UAM	Unmanned Aerial Manipulator
UAMs	Unmanned Aerial Manipulators
UAV	Unmanned Aerial Vehicle
UAVs	Unmanned Aerial Vehicles
2D	2-dimension
3D	3-dimension
SLAM	Simultaneous Localization and Mapping
YOLO	You Only Look Once
PLA	Poly Lactic Acid
ESC	Electronic Speed Controller
VPIB	Variable Parameter Integral Backstepping
IBVS	Image-Based Visual Servo
PID	Proportional Integral Derivative
ENU	East, North, and Up
D-H	Denavit-Hartenberg
CoG	Center of Gravity
ToF	Time of Flight
DoF	Degree of Freedom
ROS	Robot Operating Syste
VIO	Visual Inertial Odometry
EKF	Extended Kalman Filter
GPS	Global Positioning System
EKF	Extended Kalman Filter
IMU	Inertial Measurement Unit
FPS	Frames Per Second
CFD	Computational Fluid Dynamics
IK	Inverse Kinematics
RRPN	Rotation Region Proposal Networks
RPN	Region Proposal Network
RoIs	Region of Interests
R-anchors	Rotation anchors
IoU	Intersection over Union
AP	Average Precision
NMS	Non-maximum Suppression
CPU	Central Processing Unit
GPU	Graphics Processing Unit

## References

[1] Lendzioch T., Langhammer J., Jenicek M. (2019). Estimating Snow Depth and Leaf Area Index Based on UAV Digital Photogrammetry. Sensors.

[2] Olivares-Mendez M.A., Fu C., Ludivig P., Bissyandé T.F., Kannan S., Zurad M., Annaiyan A., Voos H., Campoy P. (2015). Towards an Autonomous Vision-Based Unmanned Aerial System against Wildlife Poachers. Sensors.

[3] Ruggiero F., Lippiello V., Ollero A. (2018). Aerial Manipulation: A Literature Review. IEEE Robot. Autom. Lett..

[4] Kim S., Choi S., Kim H.J. Aerial manipulation using a quadrotor with a two DOF robotic arm. Proceedings of the 2013 IEEE/RSJ International Conference on Intelligent Robots and Systems (IROS).

[5] Korpela C., Orsag M., Danko T., Oh P. Insertion tasks using an aerial manipulator. Proceedings of the 2014 IEEE International Conference on Technologies for Practical Robot Applications (TePRA).

[6] Božek P., Al Akkad M.A., Blištan P., Ibrahim N.I. (2017). Navigation control and stability investigation of a mobile robot based on a hexacopter equipped with an integrated manipulator. Int. J. Adv. Robot. Syst..

[7] Arleo G., Caccavale F., Muscio G., Pierri F. Control of quadrotor aerial vehicles equipped with a robotic arm. Proceedings of the 21st Mediterranean Conference on Control and Automation (MED).

[8] Jimenez-Cano A., Martin J., Heredia G., Ollero A., Cano R. Control of an aerial robot with multi-link arm for assembly tasks. Proceedings of the 2013 IEEE International Conference on Robotics and Automation (ICRA).

[9] Ruggiero F., Trujillo M.A., Cano R., Ascorbe H., Viguria A., Peréz C., Lippiello V., Ollero A., Siciliano B. A multilayer control for multirotor UAVs equipped with a servo robot arm. Proceedings of the 2015 IEEE International Conference on Robotics and Automation (ICRA).

[10] Ohnishi Y., Takaki T., Aoyama T., Ishii I. Development of a 4-joint 3-DOF robotic arm with anti-reaction force mechanism for a multicopter. Proceedings of the 2017 IEEE/RSJ International Conference on Intelligent Robots and Systems (IROS).

[11] Fabra F., Zamora W., Masanet J., Calafate C.T., Cano J.C., Manzoni P. (2019). Automatic system supporting multicopter swarms with manual guidance. Comput. Electr. Eng..

[12] Barka E., Kerrache C., Hussain R., Lagraa N., Lakas A., Bouk S. (2018). A Trusted Lightweight Communication Strategy for Flying Named Data Networking. Sensors.

[13] Kim S., Seo H., Choi S., Kim H.J. (2016). Vision-Guided Aerial Manipulation Using a Multirotor With a Robotic Arm. IEEE/ASME Trans. Mech..

[14] Ramon Soria P., Arrue B.C., Ollero A. (2017). Detection, location and grasping objects using a stereo sensor on UAV in outdoor environments. Sensors.

[15] Kanellakis C., Terreran M., Kominiak D., Nikolakopoulos G. On vision enabled aerial manipulation for multirotors. Proceedings of the 22nd IEEE International Conference on Emerging Technologies and Factory Automation (ETFA).

[16] Ren S., He K., Girshick R., Sun J. Faster r-cnn: Towards real-time object detection with region proposal networks. Proceedings of the Advances in Neural Information Processing Systems 28: 29th Annual Conference on Neural Information Processing Systems 2015.

[17] Redmon J., Divvala S., Girshick R., Farhadi A. You only look once: Unified, real-time object detection. Proceedings of the 2016 IEEE Conference on Computer Vision and Pattern Recognition (CVPR).

[18] Wu B., Wan A., Iandola F., Jin P.H., Keutzer K. SqueezeDet: Unified, Small, Low Power Fully Convolutional Neural Networks for Real-Time Object Detection for Autonomous Driving. Proceedings of the 2017 IEEE Conference on Computer Vision and Pattern Recognition (CVPR).

[19] Iandola F.N., Han S., Moskewicz M.W., Ashraf K., Dally W.J., Keutzer K. (2016). SqueezeNet: AlexNet-level accuracy with 50x fewer parameters and <0.5MB model size. arXiv.

[20] Krizhevsky A., Sutskever I., Hinton G.E. ImageNet classification with deep convolutional neural networks. Proceedings of the 25th International Conference on Neural Information Processing Systems.

[21] Ma J., Shao W., Ye H., Wang L., Wang H., Zheng Y., Xue X. (2018). Arbitrary-Oriented Scene Text Detection via Rotation Proposals. IEEE Trans. Multimed..

[22] Yang X., Sun H., Fu K., Yang J., Sun X., Yan M., Guo Z. (2018). Automatic ship detection in remote sensing images from google earth of complex scenes based on multiscale rotation dense feature pyramid networks. Remote Sens..

[23] Qin T., Li P., Shen S. (2018). Vins-mono: A robust and versatile monocular visual-inertial state estimator. IEEE Trans. Robot..

[24] Siciliano B., Sciavicco L., Villani L., Oriolo G. (2010). Robotics: Modelling, Planning And Control.

[25] Quigley M., Conley K., Gerkey B., Faust J., Foote T., Leibs J., Wheeler R., Ng A.Y. ROS: An open-source Robot Operating System. Proceedings of the ICRA Workshop on Open Source Software.

[26] Delmerico J., Scaramuzza D. (2018). A Benchmark Comparison of Monocular Visual-Inertial Odometry Algorithms for Flying Robots. Memory.

[27] Rehder J., Nikolic J., Schneider T., Hinzmann T., Siegwart R. Extending kalibr: Calibrating the extrinsics of multiple IMUs and of individual axes. Proceedings of the 2016 IEEE International Conference on Robotics and Automation (ICRA).

[28] Diaz P.V., Yoony S. High-Fidelity Computational Aerodynamics of Multi-Rotor Unmanned Aerial Vehicles. Proceedings of the 2018 56th AIAA Aerospace Sciences Meeting.

[29] Tripathi S., Dane G., Kang B., Bhaskaran V., Nguyen T. LCDet: Low-Complexity Fully-Convolutional Neural Networks for Object Detection in Embedded Systems. Proceedings of the 2017 IEEE Conference on Computer Vision and Pattern Recognition Workshops (CVPRW).

[30] Xie L., Ahmad T., Jin L., Liu Y., Zhang S. (2018). A New CNN-Based Method for Multi-Directional Car License Plate Detection. IEEE Trans. Intell. Transp. Syst..

[31] Redmon J., Farhadi A. YOLO9000: Better, Faster, Stronger. Proceedings of the 2017 IEEE Conference on Computer Vision and Pattern Recognition (CVPR).

[32] Wang J., Guo W., Pan T., Yu H., Duan L., Yang W. Bottle Detection in the Wild Using Low-Altitude Unmanned Aerial Vehicles. Proceedings of the 2018 21st International Conference on Information Fusion (FUSION).

[33] Abadi M., Barham P., Chen J., Chen Z., Davis A., Dean J., Devin M., Ghemawat S., Irving G., Isard M. Tensorflow: A system for large-scale machine learning. Proceedings of the 12th USENIX Symposium on Operating Systems Design and Implementation (OSDI).

